# Open questions: are the dynamics of ecological communities predictable?

**DOI:** 10.1186/1741-7007-12-22

**Published:** 2014-04-04

**Authors:** H Charles J Godfray, Robert M May

**Affiliations:** 1 Department of Zoology, South Parks Road, Oxford OX1 3PS, UK

## 

Einstein famously said that the ‘most incomprehensible thing about the universe is that it is comprehensible’ [1]. Perhaps the key question in ecology today is the degree to which the dynamics of ecological communities are comprehensible.

All ecological communities are made up of large numbers of species that interact in myriad ways with each other. They can be conceptualized as dynamic systems where it may be necessary to describe the spatial location, age, sex, genotype or other properties (states) of the individuals that comprise populations of different species [2]. How a species’ density and state distribution change over time is determined by complex and often poorly understood functions of that (and other) species’ densities and states. Evolutionary processes ultimately shape these functions. The historical and contingent nature of Darwinian evolution leads to a bewildering variety of forms of interaction, far beyond those observed in complex non-biological systems. Evolution also means that the functions and parameters that describe the system’s behavior may change over time so that the ecological dynamics are embedded in a larger arena of evolutionary dynamics [3].

Ecologists want to understand many things about how natural communities came to be as they are. An increasingly pressing concern is to be able to say what happens when communities experience a perturbation. Will the community dampen or amplify the perturbation, or will profound regime change occur? Are there general rules that will guide our stewardship of natural communities and allow us to protect the services they provide us [4]?

Faced with this complexity and the impossibility of ever being able to describe completely the underlying dynamics, ecologists have pursued several strategies, some with great success. The obvious approach is to simplify the problem, which can be done in different ways.

If a particular set of species interact more strongly with each other than with the rest of the community, then it may be possible to abstract and analyze their joint dynamics. This is classical single and multiple species population dynamics, which has given us unparalleled insights into many ecological interactions, and lies at the core of much applied ecology involving conservation, resource management and epidemiology [5]. It is currently enjoying a renaissance spurred by advances in linking modeling with data (for example, [6]). Nevertheless, this approach tends to run into the sands as the number of species becomes more than a handful. One possible exception is when the behavior of a whole ecosystem is critically dependent on the dynamics of one or a few ‘keystone’ species. A classic example of this is the importance of predatory starfish (Figure 1) in maintaining diversity in north-west American coastal communities [7]. But often the role of keystone species is recognized only after a perturbation.

**Figure 1:**
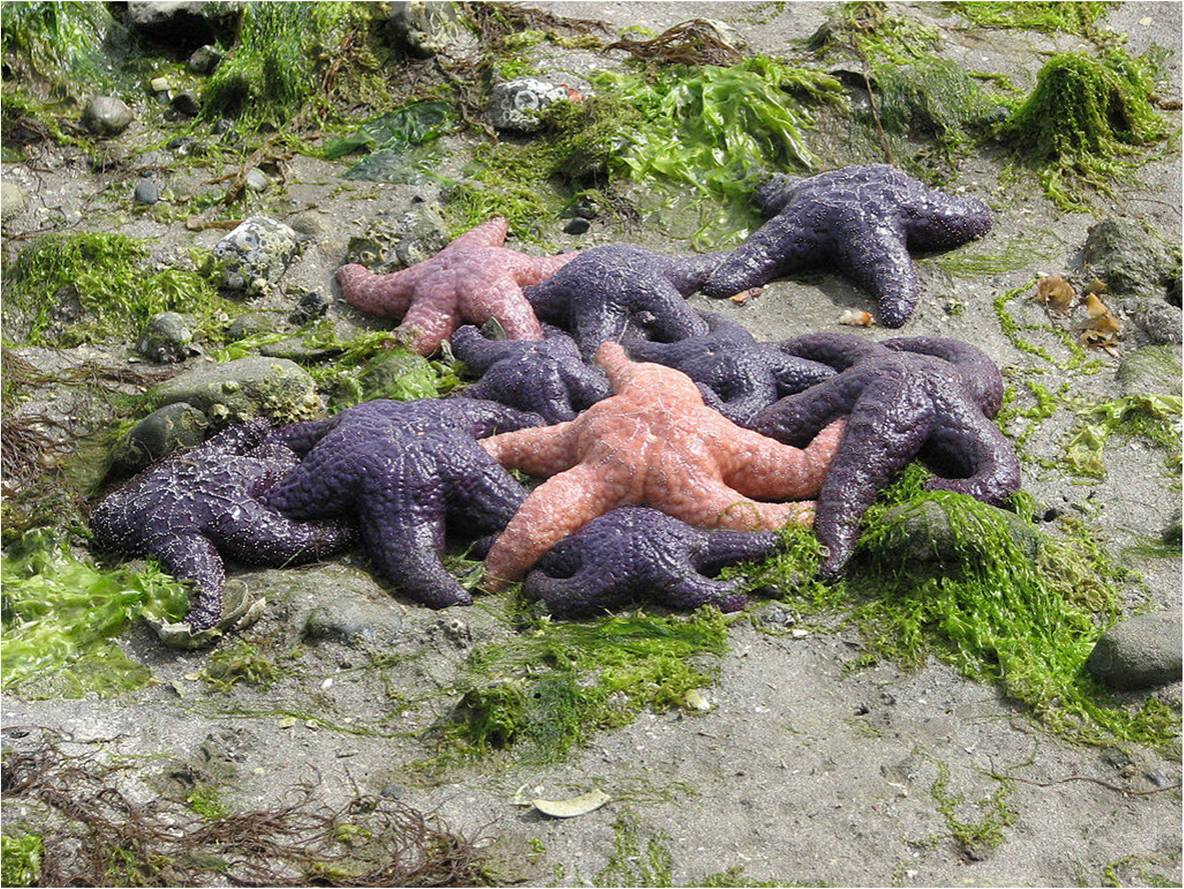
***Pisaster ochraceus *is a keystone predator in intertidal communities in the American north-west.** In a classic experiment Robert Paine removed starfish from the shoreline and found the communities became much less diverse and dominated by a single species of mussel (*Mytilus californianus*). Image credit: D. Gordon E. Robertson.

A second tactic is to make simplifications about how species interact. For example, an assemblage of species might be assumed to interact solely through competition for resources. This has been particularly successful in plant ecology because of the common basic requirements for plant growth - sunlight, water, a limited set of nutrients [8]. Using multispecies forest models, the possible responses to perturbations, such as climate change, can be explored [9]. But does it matter omitting the other components of the community: the mycorrhizal fungi often essential to nutrient acquisition, plant diseases and large and small herbivores. Taking this approach to extremes, neutral models of plant communities assume all individuals of all species are equivalent and interact with each other to the same extent [10]. Despite this gross simplification, some of the macroecological patterns produced by these models (for example, species-abundance distributions) are surprisingly close to those observed in nature [11], suggesting that they arise from the general statistics of assemblages rather than being of biological origin.

If a system is near to equilibrium, it is reasonable to assume that the dynamics of each species can be described as linear functions of the densities and states of other species. Linearization is a very powerful mathematical tool that has given great insights into community structure - for example, showing that the intuitive link between complexity and system stability is not necessarily true [12]. It can potentially address the perturbation question, but only for systems near equilibrium subject to relatively mild perturbation. Mathematical theory exists, in principle, for exploring broader classes of perturbation though in practice the complexity of biological systems frustrates deep analytical insights.

A final simplification is to lump species together into functional groups, or to dispense with species altogether and model the fluxes of energy, carbon, water or other quantity [13,14]. For example, different plant species may simply be characterized as herbs, forbs, trees, or nitrogen-fixers, or more complex classifications can be employed [15]. Again, this can give important insights, though such models also become complex quite quickly. They also cannot address questions of how perturbations affect biodiversity - a tree monoculture may have similar carbon stocks and flows to a much more diverse forest - nor do they easily lend themselves to answering evolutionary questions.

Simplifications and assumptions, what is sometimes (and slightly sneeringly) called the reductionist agenda, have delivered powerful insights into many aspects of ecological dynamics. But they have not provided a general theory of how ecological communities respond to perturbation. Are there alternatives?

Complexity theory is a portmanteau term for a diverse collection of mathematical results and conjectures on complex systems [16-21]. It studies systems that exist far from equilibrium and whose intrinsic dynamics are typically both chaotic and high dimensional. It asks whether their dynamics typically come to occupy a subset of possible system states or configurations (sometimes described as self-organization). This is a rich and interesting subject that often takes its inspiration from biological systems. It is still a young and occasionally flaky field that perhaps in ecology has so far been more productive at generating interesting think pieces and metaphors than deep insights into how biological communities actually behave. Nevertheless, it is one of the most likely sources of new ideas and innovation to increase our understanding of the resilience of ecological systems.

Ecology is a young subject. The British Ecological Society, which is the world’s oldest ecological organization, celebrated its centenary only last year, and the American Ecological Society does so next year. The first half-century focused, sensibly enough, on cataloguing the variety of plants and animals found in different regions, and on codifying the differences among them. More recent work focuses on the whys and wherefores of these differences.

Such research differs in an important way from most other areas of science, in that ever-increasing numbers of humans, each on average with a greater ‘ecological footprint’, are having serious impacts on natural ecosystems. We are in critical need of a better understanding of how communities of plants and animals put themselves together, and how the ecological services they deliver - not accounted for in conventional economics metrics such as gross domestic product (GDP) - are likely to be affected by habitat destruction, introduced aliens and many other consequences of human activity. This understanding must then be coupled with new social-science and economic thinking to craft policies to enable humans to flourish in an environment where substantial natural biodiversity is allowed to survive.

## Competing interests

The authors declare that they have no competing interests.
